# Estimation of Human Arm Joints Using Two Wireless Sensors in Robotic Rehabilitation Tasks

**DOI:** 10.3390/s151229818

**Published:** 2015-12-04

**Authors:** Arturo Bertomeu-Motos, Luis D. Lledó, Jorge A. Díez, Jose M. Catalan, Santiago Ezquerro, Francisco J. Badesa, Nicolas Garcia-Aracil

**Affiliations:** Neuro-Bioengineering Research Group, Miguel Hernandez University, Avda. de la Universidad W/N, 03202 Elche, Spain; llledo@umh.es (L.D.L.); jdiez@umh.es (J.A.D.); jose.catalan@goumh.umh.es (J.M.C.); sezquerro@umh.es (S.E.); fbadesa@umh.es (F.J.B.); nicolas.garcia@umh.es (N.G.-A.)

**Keywords:** kinematic reconstruction, neuro-rehabilitation, end-effector robots, upper limbs, MARG

## Abstract

This paper presents a novel kinematic reconstruction of the human arm chain with five degrees of freedom and the estimation of the shoulder location during rehabilitation therapy assisted by end-effector robotic devices. This algorithm is based on the pseudoinverse of the Jacobian through the acceleration of the upper arm, measured using an accelerometer, and the orientation of the shoulder, estimated with a magnetic angular rate and gravity (MARG) device. The results show a high accuracy in terms of arm joints and shoulder movement with respect to the real arm measured through an optoelectronic system. Furthermore, the range of motion (ROM) of 50 healthy subjects is studied from two different trials, one trying to avoid shoulder movements and the second one forcing them. Moreover, the shoulder movement in the second trial is also estimated accurately. Besides the fact that the posture of the patient can be corrected during the exercise, the therapist could use the presented algorithm as an objective assessment tool. In conclusion, the joints’ estimation enables a better adjustment of the therapy, taking into account the needs of the patient, and consequently, the arm motion improves faster.

## 1. Introduction

Robot-aided neuro-rehabilitation therapies have become an interesting field in the robotics area. There are several devices, such as exoskeletons, prosthesis or end-effector configuration robots, developed for this purpose [1,2]. They are able to help and assist the shortcomings of human beings. Post-stroke patients usually lose limb mobility due to the impairment in motor activity. Rehabilitation in this field takes an important role when it comes to improving the motor and proprioceptive activity [3,4]. In terms of the activities of daily living (ADL), the total or partial recovery of the upper limbs is the most important part in early rehabilitation. End-effector configuration robots are the most common devices used in these therapies. They are easily adapted to and easy to use by patients with different diseases.

These robots provide objective information about the trajectory followed by the end effector and the improvement in the motor recovery. However, they are not able to measure and control the arm movements. The progress in the arm joints, *i.e*., the range of motion (ROM), is an important parameter in these kinds of therapies. This estimation requires non-invasive wearable sensors, which must be easy to place onto the patient’s arm and must be extended to a clinical environment. Visual feedback of the arm configuration is studied in some rehabilitation therapies, though the arm joints cannot be measured [5,6]. This estimation can be accurately performed with optoelectronic systems based on motion tracking, even though they cannot be adapted to a rehabilitation environment [7,8]. In 2006, Mihelj developed a method to estimate the arm joints through two accelerometers placed onto the upper arm [9]. Then, Papaleo *et al.* improved this method using a numerical integration through the augmented Jacobian in order to estimate the arm configuration with only one accelerometer [10,11]. This algorithm performs a kinematic reconstruction of the simplified human arm model with seven degrees of freedom (DoFs) assuming that the shoulder is fixed during the therapy. Due to the loss of motor function, shoulder movements cannot be avoided by the patient, and therefore, this assumption cannot be always accomplished. Thus, it is necessary to measure shoulder movements in order to correct the position of the patient during the activity. This compensation can be detected and categorized through the fusion of a depth camera with skeleton tracking algorithms [12]. However, to compute the kinematic reconstruction, the position and orientation of the shoulder with respect to the robot are necessary.

**Figure 1:**
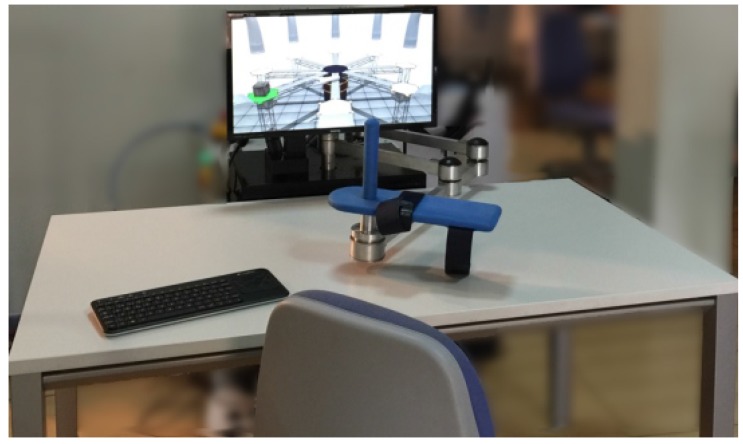
PUPArm robot.

This paper presents a kinematic reconstruction algorithm of human arm joints assuming a simplified model with five DoFs. Furthermore, this method is able to estimate the shoulder movement, *i.e*., its position and orientation. It is based on the inverse kinematics through the pseudo-inverse of the Jacobian [13]. The end-effector planar robot, called “PUPArm”, with three DoFs (see Figure 1), designed and built by Neuro-Bioengineering Research Group (nBio), Miguel Hernández University of Elche, Spain, is used [14]. The accuracy of the estimated joints with respect to the real arm joints, measured through a tracking camera, is studied. In addition, the ROMs on 50 healthy subjects performing a therapy activity are evaluated in two different cases: trying not to move the shoulder during the exercise and following the movement with the trunk to reach the goal.

## 2. Algorithm Description

### 2.1. Human Arm Kinematic Chain

**Figure 2:**
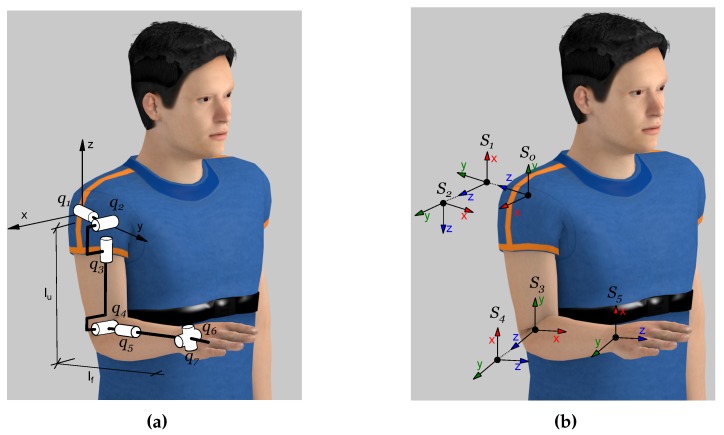
Human arm joints. (**a**) Simplification of human arm joints with seven DoFs; (**b**) Denavit–Hartenberg (DH) coordinate systems of the arm with five DoFs.

The human arm is a complex kinematic chain that can be defined as the contribution of several robotic joints. The arm was defined as a chain of nine rotational joints by Lenarčič and Umek [15]. Only seven DoFs take part in this experiment: a spherical joint in the shoulder; an elbow joint; and a spherical joint in the wrist; as is shown in Figure 2a. On the other hand, the PUPArm robot fixes two kinds of movements: the ulnar-radial deviation and the flexion-extension of the hand; thus, abduction-adduction ($q_{1}$), flexion-extension ($q_{2}$) and internal-external rotation ($q_{3}$) of the shoulder, flexion-extension ($q_{4}$) of the elbow and pronation-supination ($q_{5}$) of the forearm comprise the kinematic chain linked through two segments: the upper arm ($l_{u}$) and the forearm ($l_{f}$). The Denavit–Hartenberg (DH) parameters of the arm are shown in Table 1, and their reference systems are shown in Figure 2b.

**Table 1:** DH parameters of the kinematic arm chain.

*i*	$\theta_{i}$	$d_{i}$	$a_{i}$	$\alpha_{i}$
1	$\pi /2+q_{1}$	0	0	π/2
2	$3\pi /2+q_{2}$	0	0	π/2
3	$q_{3}$	$l_{u}$	0	−π/2
4	$\pi /2+q_{4}$	0	0	π/2
5	$q_{5}$	$l_{f}$	0	0

### 2.2. Integration Method

The inverse kinematics of the human arm during the exercise is based on the numerical integration through the pseudo-inverse of the Jacobian (*J*) [10]. The necessary devices to estimate the arm joints are: the end-effector robot; an accelerometer placed onto the upper arm and a magnetic angular rate and gravity (MARG) device placed onto the shoulder. Instantaneous joint velocities may be assessed as:

(1) $$\dot{\vec{q}}=J^{-1}\left(\vec{q}\right)\left\{\dot{\vec{v_{d}}}+K\cdot \vec{err}\right\}$$

being $\dot{\vec{v_{d}}}$ the Cartesian vector of the hand velocity and $\vec{err}$ the error committed due to the numerical integration. It should be noted that $\dot{\vec{v_{d}}}$ is the hand velocity vector with respect to the shoulder, estimated through the MARG and the accelerometer. To minimize this error, a 7×7 gain matrix *K* is added to this Equation [13]. Then, the current arm joints are computed as:

(2) $$\vec{q}\left(t_{k+1}\right)=\vec{q}\left(t_{k}\right)+\dot{\vec{q}}\left(t_{k}\right)\Delta t$$

where $\vec{q}\left(t_{k}\right)$ is the previous estimated joints, $\dot{\vec{q}}\left(t_{k}\right)$ is the joint velocity vector obtained through Equation (1) and Δt is the sampling time. On the other hand, the initial arm joints are necessary to begin the integration method; their computation is explained in Section 2.6.

### 2.3. Accelerometer Orientation

**Figure 3:**
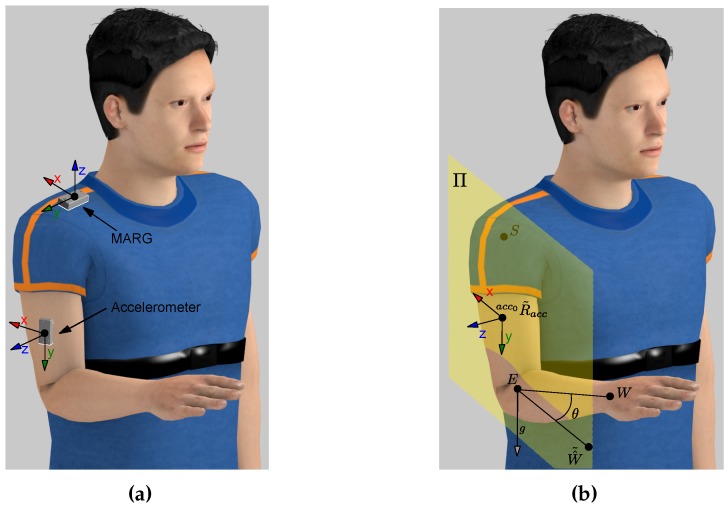
(**a**) Reference orientation of the accelerometer and the MARG. (**b**) Plane Π shaped by the *X* axis and *Y* axis of ${}^{acc_{0}}{\tilde{R}}_{acc}$.

If slow movements are assumed, the orientation of the accelerometer can be estimated in any position of the arm within the reachable workspace of the robot. When joints $q_{1}$ to $q_{5}$ are equal to zero, the reference position of the arm is set; a visual representation of this position is shown in Figure 2b. The acceleration acquired in the reference orientation of the accelerometer regarding the gravity, which is shown in Figure 3a, is:

(3) $${}^{{acc}_{0}}V_{g}=\left[\begin{array}{c} 0\\ 1\\ 0 \end{array}\right]$$

Moreover, at any random position of the arm, ${}^{{acc}_{0}}V_{g}$ can be computed through the applied rotation to the accelerometer (${}^{acc_{0}}R_{acc}$) as:

(4) $${}^{acc_{0}}V_{g}={}^{acc_{0}}R_{acc}{}^{acc}V_{g}$$

being ${}^{acc}V_{g}$ the acceleration at this random position regarding the gravity.

Equation (4) has infinite rotation matrices over the gravity vector, though one possible solution may be computed as:

(5) $${}^{acc_{0}}{\tilde{R}}_{acc}=I+M+M^{2}\frac{1-\cos\left(\theta \right)}{\sin^{2}\left(\theta \right)}$$

with:

(6) $$\begin{array}{cc} M & =\left[\begin{array}{ccc} 0 & -V_{3} & V_{2}\\ V_{3} & 0 & -V_{1}\\ -V_{2} & V_{1} & 0 \end{array}\right]\\ V & ={}^{acc_{0}}V_{g}\times {}^{acc}V_{g}\\ sin(\theta ) & =∥V∥\\ cos(\theta ) & ={}^{acc_{0}}V_{g}\cdot {}^{acc}V_{g} \end{array}$$

Thereby, a plane can be shaped by the *X* axis and *Y* axis of ${}^{acc_{0}}{\tilde{R}}_{acc}$ (plane Π). This plane only contains the elbow point (*E*), but the correct orientation of the accelerometer must also contain the shoulder (*S*) and the wrist (*W*) points. Thus, the rotation angle (*θ*) is defined as the angle between the known wrist point and the new wrist point ($\tilde{\hat{H}}$), contained in the plane Π, when it is rotated around the gravity vector (*g*) placed in *E* (see Figure 3b). Therefore, $\tilde{\hat{H}}$, expressed in terms of *θ*, can be defined as:

(7) $$\begin{array}{cc} \tilde{\hat{W}}= & \left(g\cdot \hat{W}\right)g+\cos\left(\theta \right)\left(\hat{W}-\left(g\cdot \hat{W}\right)g\right)-\sin\left(\theta \right)\left(g\times \hat{W}\right) \end{array}$$

where $\hat{W}=\left(W-E\right)/\left(∥W-E∥\right)$ and $g={\left[\begin{array}{ccc} 0 & 0 & -1 \end{array}\right]}^{T}$. Then, *θ* can be obtained solving the following equation:

(8) $$d\left(\tilde{\hat{W}},\Pi \right)=\frac{\left|A_{\Pi }{\tilde{\hat{W}}}_{x}+B_{\Pi }{\tilde{\hat{W}}}_{y}+C_{\Pi }{\tilde{\hat{W}}}_{z}+D_{\Pi }\right|}{\sqrt{{A_{\Pi }}^{2}+{B_{\Pi }}^{2}+{C_{\Pi }}^{2}}}=0$$

having the plane Π computed as follows:

(9) $$\begin{array}{cc} {\tilde{P}}_{acc}^{x} & ={}^{acc_{0}}{\tilde{R}}_{acc}{\left[\begin{array}{ccc} 1 & 0 & 0 \end{array}\right]}^{T}\\ {\tilde{P}}_{acc}^{y} & ={}^{acc_{0}}{\tilde{R}}_{acc}{\left[\begin{array}{ccc} 0 & 1 & 0 \end{array}\right]}^{T}\\ \bar{S{\tilde{P}}_{acc}^{y}} & =\left({\tilde{P}}_{acc}^{y}-S\right)\\ \bar{{\tilde{P}}_{acc}^{x}{\tilde{P}}_{acc}^{y}} & =\left({\tilde{P}}_{acc}^{y}-{\tilde{P}}_{acc}^{x}\right)\\ \left[\begin{array}{c} A_{\Pi }\\ B_{\Pi }\\ C_{\Pi } \end{array}\right] & =\bar{S{\tilde{P}}_{acc}^{y}}\times \bar{{\tilde{P}}_{acc}^{x}{\tilde{P}}_{acc}^{y}}\\ D_{\Pi } & ={\left[\begin{array}{ccc} A_{\Pi } & B_{\Pi } & C_{\Pi } \end{array}\right]}^{T}\cdot S \end{array}$$

Two possible solutions are obtained through Equation (8) and, therefore, two values of ${}^{acc_{0}}R_{acc}$. The correct solution is one for which the *Z* axis is in the same direction as the cross product between the elbow-wrist segment and elbow-shoulder segment due to the reference position of the accelerometer. Finally, the rotation of the accelerometer regarding the robot is computed as:

(10) $${}^{r}R_{acc}{=}^{r}R_{acc_{0}}\cdot^{acc_{0}}R_{acc}$$

being ${}^{r}R_{acc_{0}}$ the reference orientation of the accelerometer concerning the robot (see Figure 3a). This orientation is required to estimate the elbow orientation and shoulder position during the exercise.

### 2.4. MARG Orientation

The orientation of magneto-inertial devices is usually based on Kalman filtering [16]; nevertheless, they can be quite complicated, and an extended Kalman filter is needed to linearize the problem. The orientation filter to measure the rotation of the MARG of Madgwick *et al.* is used in this algorithm [17]. The magnetic distortion that may be introduced by external sources, including metal furniture and metal structures within a building, is performed in this filter [18]. Furthermore, the orientation algorithm requires an adjustable parameter (*β*) that can be adjusted to the requirements of this exercise. Hence, the value of this parameter (β=5) was established after a “trial and error” approach tested before the experiment, taking into account the features of the exercises.

This filter measures the reference quaternion of the device with respect to the Earth reference system, defined by the gravity vector and the Earth’s magnetic field lines. However, the rotation of the Earth concerning the robot is unknown. If the MARG is placed in a known orientation with respect to the robot (${}_{M_{0}}^{R}\hat{q}$), the acquired transformation defines the Earth frame relative to the sensor frame (${}_{E}^{M_{0}}\hat{q}$), and therefore, the reference transformation between the robot and the Earth is known as:

(11) $${}_{E}^{R}\hat{q}{=}_{M_{0}}^{R}\hat{q}{⊗}_{E}^{M_{0}}\hat{q}$$

Therefore, every rotation of the MARG is defined in the workspace as:

(12) $${}_{M}^{R}\hat{q}{=}_{E}^{R}\hat{q}{⊗}_{E}^{M}{\hat{q}}^{*}$$

where ${}_{E}^{M}\hat{q}$ is the current value of the sensor. In this way, the shoulder orientation is estimated during the exercise.

### 2.5. Elbow and Shoulder Location

The hand, as was said before, is tightly attached to the end effector of the robot, and the ulnar-radial deviation and flexion-extension of the hand remain constant. Hence, the transformation matrix between the hand and the end effector (${}^{r}T_{w}$) is known, and therefore, the elbow position may be obtained as:

(13) $${}^{r}P_{e}{=}^{r}T_{w}*{\left[\begin{array}{cccc} 0 & 0 & -l_{f} & 1 \end{array}\right]}^{T}$$

The orientation of the elbow, since the rotation matrix between the elbow and the accelerometer orientation (${}^{acc_{0}}R_{e}$) is known (see Figure 3a), may be calculated as:

(14) $${}^{r}R_{e}{=}^{r}R_{acc}\cdot^{acc_{0}}R_{e}$$

with ${}^{r}R_{acc}$ the rotation matrix computed through Equation (10). Thus, the transformation of the elbow relative to the robot remains:

(15) $${}^{r}T_{e}=\left[\begin{array}{cccc} & {}^{r}R_{e} & & {}^{r}P_{e}\\ 0 & 0 & 0 & 1 \end{array}\right]$$

On the other hand, one of the most important points of this algorithm is the ability to estimate the shoulder position and orientation during the exercise. The shoulder position can be processed easily through Equation (15) as:

(16) $${}^{r}P_{s}{=}^{r}T_{e}*{\left[\begin{array}{cccc} 0 & lu & 0 & 1 \end{array}\right]}^{T}$$

Whilst the orientation of the MARG relative to the robot is known by Equation (12), its rotation matrix ${}^{r}R_{M}$ is directly obtained [19]. Thus, the shoulder orientation is estimated as:

(17) $${}^{r}R_{s}{=}^{r}R_{M}\cdot^{M_{0}}R_{s}$$

where ${}^{r}R_{M}$ is the current rotation of the sensor with respect to the robot and ${}^{M_{0}}R_{s}$ the reference position of the MARG relative to the shoulder (see Figure 3a). Hence, the transformation of the shoulder relative to the robot remains:

(18) $${}^{r}T_{s}=\left[\begin{array}{cccc} & {}^{r}R_{s} & & {}^{r}P_{s}\\ 0 & 0 & 0 & 1 \end{array}\right]$$

Finally, since the elbow and the shoulder location are instantaneously known, the initial conditions and the integration method can be performed.

### 2.6. Initial Conditions

In this algorithm, since it is based on a numerical integration, the initial conditions are required. The locations of the three main points, namely the shoulder (${}^{r}T_{s}$), the elbow (${}^{r}T_{s}$) and the wrist (${}^{r}T_{s}$), are known. The shoulder joints ($q_{1}$, $q_{2}$ and $q_{3}$) are directly related to the matrix ${}^{s}T_{e}{=}^{r}T_{s}^{-1}\cdot^{r}T_{e}$, defined in the previous section, and they can be acquired by the spherical joint method [13]. This matrix, in terms of the corresponding joints, can be expressed by DH parameters shown in Table 1 as:

(19) $${}^{s_{0}}T_{s_{3}}={}^{s_{0}}T_{s_{1}}\cdot {}^{s_{1}}T_{s_{2}}\cdot {}^{s_{2}}T_{s_{3}}=\left[\begin{array}{cccc} c_{1}s_{3}-c_{3}s_{1}s_{2} & -c_{2}s_{1} & c_{1}c_{3}+s_{1}s_{2}s_{3} & l_{u}c_{2}s_{1}\\ s_{1}s_{3}+c_{1}c_{3}s_{2} & c_{1}c_{2} & c_{3}s_{1}-c_{1}s_{2}s_{3} & -l_{u}c_{1}c_{2}\\ -c_{2}\;c_{3} & s_{2} & c_{2}\;s_{3} & -l_{u}s_{2}\\ 0 & 0 & 0 & 1 \end{array}\right]$$

having $s_{i}=\sin\left(q_{i}\right)$ and $c_{i}=\cos\left(q_{i}\right)$, i={1,2,3}. If the transformation matrix ${}^{s_{0}}T_{s_{3}}$ is defined as:

(20) $${}^{s_{0}}T_{s_{3}}\left(q_{1},q_{2},q_{3}\right)=\left[\begin{array}{cccc} n_{x} & n_{y} & n_{z} & p_{x}\\ o_{x} & o_{y} & o_{z} & p_{y}\\ a_{x} & a_{y} & a_{z} & p_{z}\\ 0 & 0 & 0 & 1 \end{array}\right]$$

two possible solutions of the shoulder joints are obtained; if $q_{2}\in \left[0\;\pi \right]$:

(21) $$\begin{array}{cc} q_{1} & =\text{atan}2\left(-n_{y},o_{y}\right)\\ q_{2} & =\text{atan}2\left(a_{y},\sqrt{n_{y}^{2}+o_{y}^{2}}\right)\\ q_{3} & =\text{atan}2\left(a_{z},-a_{x}\right) \end{array}$$

and if $q_{2}\in \left[-\pi \;0\right]$:

(22) $$\begin{array}{cc} q_{1} & =\text{atan}2\left(n_{y},-o_{y}\right)\\ q_{2} & =\text{atan}2\left(a_{y},-\sqrt{n_{y}^{2}+o_{y}^{2}}\right)\\ q_{3} & =\text{atan}2\left(-a_{z},a_{x}\right) \end{array}$$

Thereby, the elbow joint ($q_{4}$) is directly determined with the cosine law as:

(23) $$q_{4}=\arcsin\left(\frac{l_{u}^{2}+l_{f}^{2}-{\left||H-S|\right|}^{2}}{2l_{u}l_{f}}\right)$$

and its homogeneous matrix remains:

(24) $${}^{s_{3}}T_{s_{4}}=\left[\begin{array}{cccc} -\sin\left(q_{4}\right) & 0 & \cos\left(q_{4}\right) & 0\\ \cos\left(q_{4}\right) & 0 & \sin\left(q_{4}\right) & 0\\ 0 & 1 & 0 & 0\\ 0 & 0 & 0 & 1 \end{array}\right]$$

Thus, the transformation matrix between the systems $s_{0}$ and $s_{4}$ can be computed. The known matrix ${}^{s}T_{h}{=}^{r}T_{s}^{-}1\cdot^{r}T_{h}$ defines the transformation between the system $s_{0}$ and $s_{5}$. On the other hand, the last joint, $q_{5}$, is defined with the DH parameters as:

(25) $${}^{s_{4}}T_{s_{5}}\left(q_{5}\right)=\left[\begin{array}{cccc} -\sin\left(q_{5}\right) & \cos\left(q_{5}\right) & 0 & 0\\ \cos\left(q_{5}\right) & \sin\left(q_{5}\right) & 0 & 0\\ 0 & 0 & 1 & 0\\ 0 & 0 & 0 & 1 \end{array}\right]$$

and therefore, $q_{5}$ is estimated as:

(26) $$q_{5}=\text{atan}2\left(-n_{x},o_{x}\right)$$

Finally, two possible configurations of the arm joints are found, even though only one solution is possible. Due to the limits of the arm joints, $\left[-\pi /2\;\pi /2\right]$, only one solution accomplishes this restriction, and the initial position of the arm is assessed. This method can produce abrupt changes in the estimated arm joints caused by possible perturbations in the accelerometer that might lead to a non-anatomical position. Hence, since the new position depends on the latest position and the sample time, the integration method for real-time reconstruction is the best way to overcome the aforementioned drawbacks following Equations (1) and (2).

## 3. Results and Discussion

### 3.1. Experimental Exercises

**Figure 4:**
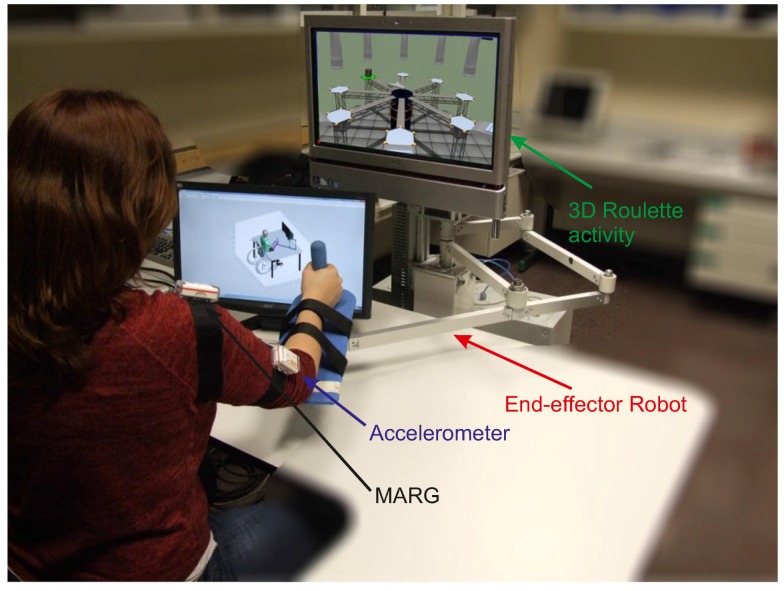
Subject wearing the sensors, the accelerometer and the MARG, grasping the end effector of the robot and performing the 3D roulette activity.

With the aim of studying the arm joint estimation algorithm, with K=diag{1.5,1.5,...1.5} N/ms (chosen by the “trial and error” approach tested before the experiment), two different experiments were performed. The first exercise was to compute the algorithm accuracy in terms of the arm joints and the position of the shoulder, performed by four healthy subjects. Then, a rehabilitation exercise with two different trials was performed by 50 healthy subject (aged between 20 and 72) to test the behavior of the presented algorithm. In both cases, the length of the upper arm was measured from the lateral side of the acromion to the proximal radius head, in the elbow joint. From the proximal radius head to the radial styloids, the distal part of the radius, the forearm length was measured [20]. Moreover, both experiments are performed under the same activity: 3D roulette, which may be seen in Figure 4. The activity consisted of taking a box from the perimeter and placing it in the center of the screen; hand movements are symbolized as a wrench (see Figure 4). One movement is considered when the subject goes from the center of the roulette to the perimeter and returns again to the center.

A magneto-inertial sensor, developed by Shimmer^©^, is tightly attached onto the upper arm and onto the shoulder to compute the kinematic reconstruction algorithm. The real position of the arm is computed with a six DoF optical tracking camera Optitrak V120: Trio, developed by NaturalPoint^®^. Specific parts attached to the hand, upper arm and forearm with retro-reflective markers were developed for this purpose. Information about the subjects who carried out the validation experiment are shown in Table 2; they performed three trials of the same exercise.

**Table 2:** Main subject data from the validation experiment.

ID	Age	Gender	Forearm Length (m)	Upper Arm
1	21	Male	0.23	0.32
2	51	Female	0.21	0.33
3	32	Male	0.25	0.31
4	31	Male	0.21	0.33

In the second experiment, two different trials of the same activity were performed. The first trial was intended not to move the shoulder while the exercise was being conducted, *i.e*., without compensation with the trunk. However, the participants were asked to follow the hand movements with the shoulder in the second exercise. Each trial consisted of 24 movements.

### 3.2. Algorithm Validation

**Figure 5:**
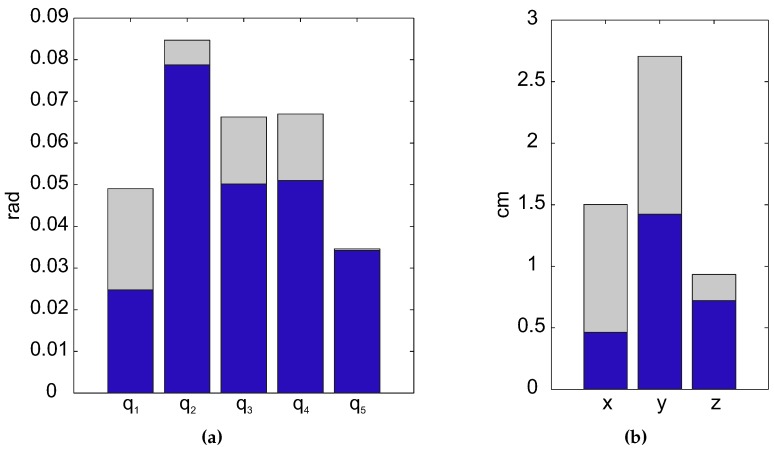
Error committed in the reconstruction algorithm. (**a**) Mean RMSE (blue bar) of the joints committed by the subjects and standard deviation (gray bar); (**b**) Mean RMSE (blue bar) of the shoulder position committed by the subjects and the standard deviation (gray bar): x, left/right movements; y, forward/backward movements; z, up/down movements.

**Figure 6:**
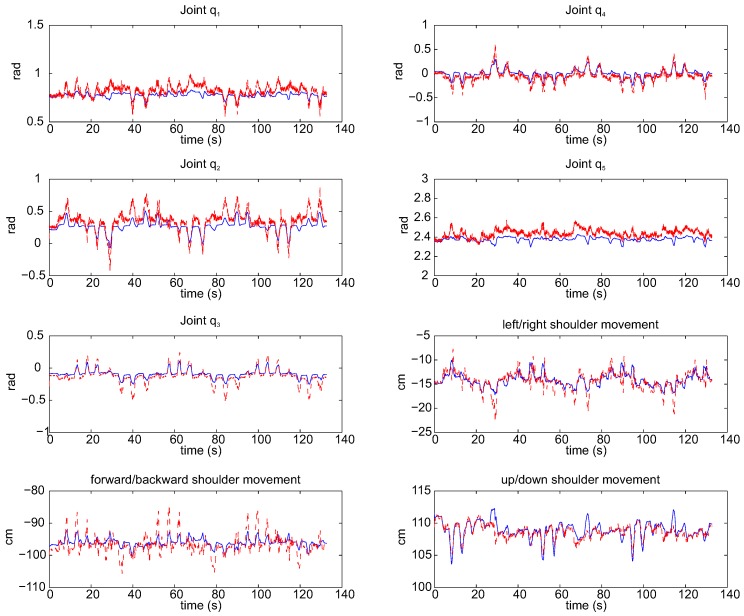
Joints and shoulder movements estimated through the algorithm (dotted red line) and measured through the optoelectronic system (blue line) of a subject during an exercise.

The mean error committed, in terms of root mean square error (RMSE) and standard deviation, is shown in Figure 5a. The mean RMSE of the joints is 0.047 rad with a standard deviation of 0.013 rad. Otherwise, the error committed on the shoulder position estimation, which may be found in Figure 5b, shows the mean RMSE committed, less than 0.87 cm, and the standard deviation, around 0.83 cm. The good results show that the error committed is small (it is hardly noticeable by the human eye), and therefore, the accuracy of the presented algorithm with respect to the real arm movements is high. A kinematic reconstruction of the arm joints and the estimation of shoulder position acquired from both methods through the presented algorithm (red dotted line) and the direct reconstruction (blue line) are pictured in Figure 6.

### 3.3. Arm Joint Range

In this experiment, the ROM between both trials, with and without compensation with the trunk, is studied. Furthermore, the shoulder movement is compared to its real position, acquired with the optoelectronical system mentioned before. To compare both groups, statistical analysis is performed through the *t*-test for paired data for each ROM. Joints 1 to 4 show significant differences (p≤0.05), but nevertheless, Joint 5, as the subject wrist is attached to the end effector of the robot, does not show significant differences (p=0.064).

**Figure 7:**
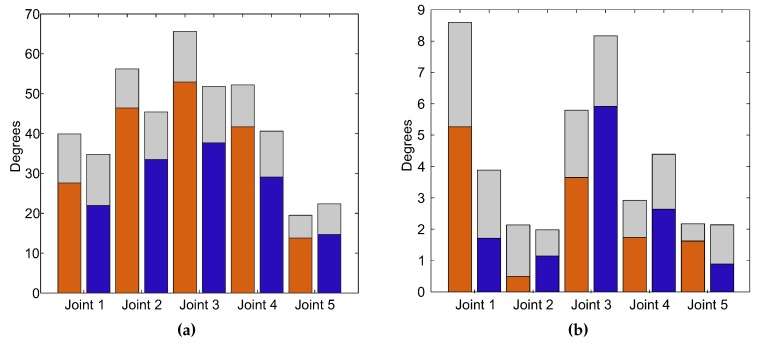
Representation of both trials: without compensation (orange bar) and with compensation (blue bar), and the standard deviation (gray bar) in terms of arm joints. (**a**) Estimated range of motion (ROM); (**b**) Error committed between the real ROM and the estimated ROM.

The estimated ROM in the exercise without compensation and with compensation is shown in Figure 7a, and the error committed might be seen in Figure 7b. It should be noted that the error committed in each joint for both exercises is smaller than six degrees. On the other hand, the ROM estimated for the trial without compensation is larger than that from the other trial. This result was expected, because the shoulder compensation affects the joint range. However, the ROMs of Joint 5 are similar, because the pronation-supination of the forearm is not affected when the compensation is performed.

**Figure 8:**
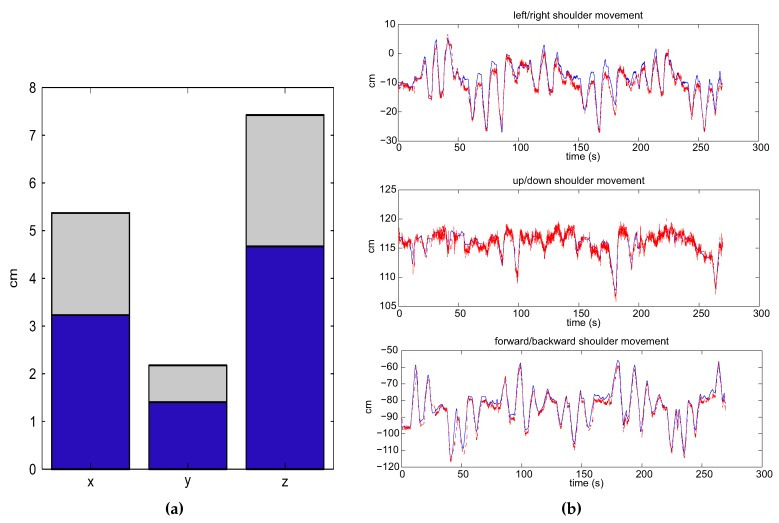
Shoulder movement in the compensation trial. (**a**) Mean RMSE (blue bar) committed by the population and the standard deviation (gray bar): x, left/right movements; y, forward/backward movements; z, up/down movements; (**b**) Estimated movement through the proposed algorithm (dotted red line) and the direct movement (blue line) performed by one subject.

The accuracy of the shoulder position, taking into account the whole population (N = 51), is shown in Figure 8a. The estimated shoulder position with respect to the real shoulder location in a compensation trial performed by one subject can be seen in Figure 8b.

## 4. Conclusions

In this paper, a kinematic reconstruction of the upper limbs during robot-aided rehabilitation with planar robots taking into account shoulder movements is presented. The estimated arm joints are very accurate with respect to the real position of the arm. Thus, the arm joint improvements of the patient can be measured objectively, and a better adaptation of the therapy to the patient needs can be also performed.

The measurement of the shoulder movement can be also computed accurately. To the best of our knowledge, this feature is not included in the previous algorithms where the shoulder is assumed to be fixed, even when little movements cannot be avoided during the exercise. This feature helps the therapist to correct the patient’s posture during exercise for faster improvement in terms of arm mobility.

In summary, the arm joints’ improvement may be included as a new objective assessment parameter in addition to the motor and proprioceptive activity and assessments scales, which are, by definition, subjective, as the Fugl–Meyer assessment [21].
